# Bronchial Asthma Exacerbation in the Emergency Department in a Saudi Pediatric Population: An Insight From a Tertiary Hospital in Riyadh, Saudi Arabia

**DOI:** 10.7759/cureus.33391

**Published:** 2023-01-05

**Authors:** Mohammed A Alfurayh, Mouath A Alturaymi, Ahmed Sharahili, Majed A Bin Dayel, Abdullah I Al Eissa, Muath O Alilaj

**Affiliations:** 1 Medicine and Surgery, King Saud Bin Abdulaziz University for Health Sciences College of Medicine, Riyadh, SAU; 2 Family Medicine, King Abdulaziz Medical City Riyadh, Riyadh, SAU

**Keywords:** rhinitis, exacerbation, eczema, allergy, asthma

## Abstract

Background

Severe uncontrolled asthma in the pediatric population is a complicated disease and is considered a major challenge for pediatricians. Severe bronchial asthma in the pediatric population is related to significant morbidity and mortality. Children with complicated asthma are at a higher risk for unfavorable outcomes, including medication-associated adverse effects, severe life-threatening exacerbations, and poor quality of life.

Methodology

A cohort study was conducted at National Guard Health Affairs Hospital in Riyadh, Saudi Arabia. Data were collected using the chart review method utilizing a data collection sheet. A total of 363 charts of children aged one month to 14 years who visited the emergency room (ER) due to asthma exacerbation at NGHA were reviewed, from January 2016 to May 2022, to extract the variables. Variables included demographic data, comorbidities, and asthma-related variables which included the number of asthma exacerbations, hospital admission, ER visit, medication use (non-steroidal anti-inflammatory drugs and steroids), and the presence of allergic rhinitis and eczema.

Results

A total of 363 patients were analyzed, with 229 (63.1%) males and 134 (36.9%) females. The mean age was 4.9 years (SD = 3.5 years). Overall, 8.5% of patients had congenital heart disease, 4.1% had gastroesophageal reflux disease, 2.2% had diabetes mellitus, 1.9% had obstructive sleep apnea, and 0.6% had hypertension. Most patients presented with a cough at 88.2% (n = 320), followed by shortness of breath at 59% (n = 214) and fever at 46% (n = 167). Male asthmatics visited ER more than females. Forty-four patients were admitted to the hospital. Inhaled steroids were associated with fewer emergency department visits and night symptoms. Most asthmatic patients presented in the winter and fall seasons.

Conclusions

Asthma is a common pediatric respiratory disease that could be a burden if not controlled well. Unfortunately, the frequency of hospital admissions and pediatric ER visits due to asthma exacerbation is increasing. Comorbidities such as obesity and gastroesophageal reflux disease play a significant role in asthma control.

## Introduction

Bronchial asthma is the most commonly reported pediatric chronic respiratory disease, as reported in multiple previous studies [1]. Despite the dramatic development that is seen in primary healthcare centers, asthma in children remains a global burden with high costs. Airway obstruction in bronchial asthma presents in variable intensities ranging from mild to more severe forms [2]. Bronchial asthma is subdivided into two common subtypes, namely, allergic and non-allergic asthma [3]. Non-allergic asthma is not that common compared to allergic asthma. Allergic asthma usually co-exists with other atopic clinical presentations such as allergic rhinitis and eczema [4]. Clinical presentation and serum immunoglobulin E (IgE) levels are diagnostic tools that are used to distinguish between allergic and non-allergic asthma [5]. Moreover, interactions between the affected person’s gene and the surrounding environmental causes increase the person’s susceptibility to developing the allergic type of asthma [6]. Patients with asthma can experience worsening symptoms that make them seek medical attention, a phenomenon called asthma exacerbation. It can range from mild to more severe life-threatening exacerbations [7]. Exacerbation tends to increase in frequency with asthma severity. Severe asthma is known to be associated with more frequent exacerbations with higher costs [8]. An exacerbation is often the first symptom leading to the diagnosis of bronchial asthma and constitutes the vast majority of emergency department (ED) visits [9].

In the United States, multiple studies have shown a significant difference regarding the prevalence of asthma in multiple states. It is believed that this discrepancy is best explained by the different environments and weather [10]. In the Middle East, asthma prevalence has been investigated in many countries; however, poor documentation has been observed in almost all countries except Iran and Saudi Arabia. In Saudi Arabia, the prevalence of asthma has been reported by many studies; however, different age groups were assessed, and different tools were employed to assess asthma in Saudi Arabia. The highest prevalence was estimated in Alhofuf and the lowest in the Abha region [11]. Additionally, two separate studies conducted in Jazan and Taif in Saudi Arabia reported that asthma was more prevalent in rural areas when compared to urban areas [12]. The prevalence of allergic rhinitis in the Middle East was documented to be 9-38%, according to a study done in Jeddah, Saudi Arabia [13].

Around 5% of the pediatric population have severe asthma, which is characterized by uncontrolled asthma symptoms despite providing optimal care and treatment [14]. Around one-half of children with asthma present with asthma exacerbation symptoms which can be caused by a variety of risk factors, such as dust, pets, viral upper respiratory tract infection (URTI), smoking, exercise, and obesity, among other risk factors [15]. Furthermore, in Saudi Arabia, it has been shown that a high body mass index (BMI) is associated with asthma in pre-pubertal Saudi boys and girls [16]. In addition, severe asthma and obesity have been related to frequent emergency room (ER) visits, as documented in one study [17]. Nevertheless, a study from the United States showed that environmental tobacco smoking is associated with a significantly high risk for asthma in the pediatric population. Thus, smoking around children in the house or in public areas is a major risk factor for worsening asthma symptoms [18].

Uncontrolled asthma is the most common risk factor for asthma exacerbation. Thus, uncontrolled asthma is one of the most common causes of hospital admissions and ER visits. Furthermore, the significant rise in the number of hospital admissions because of asthma may reflect an increase in asthma severity, poor asthma management, limited access to primary care, and/or the effect of poverty [19]. On the other hand, countries that have developed asthma management strategies have observed decreases in hospital admission rates due to asthma [20]. Because there are a few published studies from Saudi Arabia concerning the variation in factors related to asthma exacerbation among the Saudi pediatric population, we aim to explore the variations in the factors affecting asthma exacerbations and other atopic diseases. We also aim to report the average number of hospital admissions and ER visits in a Saudi healthcare facility.

## Materials and methods

A cross-sectional study was conducted in the pediatric emergency department at the National Guard Health Affairs Hospital (NGHA) in Riyadh, Saudi Arabia. NGHA is an internationally recognized healthcare organization providing a wide range of clinical, academic, and research programs ranging from public health and primary care to fine tertiary care specialties and subspecialties. Data were collected electronically using the chart review method utilizing a data collection sheet. Charts of children with asthma who visited the ED were reviewed to extract the variables. Variables included age, comorbidities (diabetes mellitus (DM), hypertension (HTN), obstructive sleep apnea (OSA), and congenital heart diseases (CHD)), the number of asthma exacerbations based on ER visits, hospital admission, medications (non-steroidal anti-inflammatory drugs and steroids), and the presence of atopic diseases (allergic rhinitis and eczema).

The study included Saudi pediatric patients who met the inclusion criteria from January 2016 to May 2022. The inclusion criterion included Saudi kids aged from one month to 14 years who visited the ER with asthma exacerbation, while the exclusion criterion was all Saudi pediatric patients with other forms of bronchial asthma such as hyperactive airway disease. The sampling technique was probability simple random sampling.

Data were analyzed with both descriptive statistics, including frequency and percentage of the age category, gender of the patients, and inferential statistics to find associations between different variables with asthma. There was a statistically significant association between tested variables if the p-value was <0.05. All data were analyzed using SPSS version 27.0.1 (IBM Corp., Armonk, NY, USA).

The sociodemographic characteristics frequency, percentage, mean, and SD of asthma patients were analyzed, and the results were presented in tables and graphs. The chi-square test (when less than 20% of cells had expected frequencies <5) and Fisher’s exact test (when more than 20% of cells had expected frequencies <5) were applied to find the association between different variables and asthma.

## Results

The descriptive statistics of 363 patients were analyzed, of whom 229 (63.1%) were males and 134 (36.9%) were females (Table 1). The mean age of 363 patients was 4.9 years (SD = 3.5 years), with a minimum age of two months and a maximum age of 168 months (14 years) for a range of 166 months (13.8 years). Most of our patients were aged three to four years (35.3%, n = 128), of whom 68.8% (n = 88) were males and 31.3% (n = 40) were females, followed by the age group of fewer than two years (21.8%, n = 79), with a male and female distribution of 63% (n = 50) and 37% (n = 29), respectively. Further age groups of asthmatic patients are shown in Table 1. Furthermore, 8.5% of patients had CHD, 4.1% had gastroesophageal reflux disease (GERD), 2.2% had DM, 1.9% had OSA, and 0.6% had HTN. The frequency of different comorbidities is shown in Table 1.

**Table 1:** Descriptive statistics of different parameters of asthma patients. DM = diabetes mellitus; HTN = hypertension; GERD = gastroesophageal reflux disease; OSA = obstructive sleep apnea; CHD = congenital heart disease; SOB = shortness of breath; NSAIDs = non-steroidal anti-inflammatory drugs; URTI = upper respiratory tract infection

Parameter			Frequency (n = 363)	Percentage
Demographics	Gender	Male	229	63.1%
		Female	134	36.9%
	Age of Patients	<2 years	79	21.8%
		2–6 years	187	51.6%
		7–10 years	52	14.3%
		11–14 years	45	12.4%
Comorbidities	DM	Present	8	2.2%
	HTN	Present	2	0.6%
	GERD	Present	15	4.1%
	OSA	Present	7	1.9%
	CHD	Present	31	8.5%
Presenting symptoms	Fever	Present	167	46.0%
	Cough	Present	320	88.2%
	SOB	Present	214	59.0%
	Runny nose	Present	31	8.5%
	Sore throat	Present	10	2.8%
	Vomiting	Present	23	6.3%
Previous medical history	Medication type	Aspirin	3	0.8%
		NSAIDs	11	3.0%
		None	349	96.1%
	Steroid use	Yes	123	33.9%
	Allergen	Yes	29	8.0%
	Night symptoms	Yes	55	15.2%
	Previous asthma exacerbation	Yes	179	49.3%
	Family history of asthma	Yes	76	20.9%
	Eczema	Yes	53	14.6%
	URTI	Yes	324	89.3%
	Allergic rhinitis	Yes	26	7.2%
Seasonal variations	Summer	Yes	34	9.4%
	Winter	Yes	133	36.6%
	Fall	Yes	129	35.5%
	Spring	Yes	63	17.4%

Additionally, most asthmatic patients presented with a cough (88.2%, n = 320), followed by shortness of breath (59%, n = 214), and fever (46%, n = 167). Further associated symptoms, frequency, and percentage are shown in Table 1. Most asthmatic patients had a previous history of URTI (89.3%, n = 324), previous asthma exacerbation (49%, n = 179), and steroid use (34%, n = 123). Overall, 8% (n = 29) of patients had a previously documented history of allergy either to food, drug, or other environmental allergens; 21% of patients had a family history of asthma; and 15% of patients had night symptoms, as shown in Table 1. In addition, Table 1 shows that most of our patients (36.6%, n = 133) had asthma exacerbation in the winter followed by 35.5% (n = 129) in the fall.

Moreover, 51% (n = 185) of patients visited the ED for an asthma attack, 17.4% (n = 63) visited the ED two times, 8.8% (n = 32) visited the ED three times, and 22.9% (n = 83) visited the ED more than three times. Of these patients, 90.6% (n = 329) stayed for one day in the ED, and 9.4% patients stayed for two days in the ED, as shown in Table 2. Out of 363 patients, 11.8% (n = 43) patients were admitted to the hospital for further treatment, of whom 62.8% (n = 27) were males and 37% (n = 16) were females. Out of the 43 patients, 34 (79.1%) patients were admitted only one time, four (9.3%) were admitted two times due to asthma, and five (11.6%) were admitted three or more times. Out of the 43 admitted patients, 37.2% (n = 16) were admitted for one day, 25.6% (n = 11) were admitted for two days, and 37.2% (n = 16) were admitted for three or more days. Overall, 41% of patients had a previous history of neonatal/pediatric intensive care unit admission, as shown in Table 2.

**Table 2:** Descriptive statistics of ER visits and hospital admissions of asthma patients. ER = emergency room; NICU = neonatal intensive care unit; PICU = pediatric intensive care unit

Parameter			Frequency (n = 363)	Percentage
Hospital admissions and ER visits	Number of ER visits (during the study period)	One visit	185	51.0%
		Two visits	63	17.4%
		Three	32	8.8%
		More than three visits	83	22.9%
	Length of stay in ER	1 day	329	90.6%
		2 days	34	9.4%
	Admission to hospital	Yes	44	12.1%
		No	319	87.9%
	Number of admissions (during the study period)	1	34 (43)	79%
		2	4 (43)	9.3%
		3	5 (43)	11.6%
	Duration of hospital admission	1 day‎	16 (43)	37.2%
		2 days	11 (43)	25.6%
		3–28 days	16 (43)	37.2%
	NICU/PICU	Yes	149	41.0%
		No	214	59.0%

Overall, 44 patients were admitted to the hospital, of whom 19 asthmatic patients had other comorbidities as well. Out of the 19 admitted patients, three had DM, but the effect of DM on the admission of asthmatic patients in the hospital was not significant (p = 0.060) (Table 3). Out of the 19 admitted patients, two had HTN, and the effect of HTN on the admission of asthmatic patients in the hospital was significant (p = 0.014) (Table 3). Out of the 19 admitted patients, six had GERD, and the effect of GERD on the admission of asthmatic patients in the hospital was slightly significant (p = 0.05) (Table 3). Out of the 19 admitted patients, two had OSA, but the effect of OSA on the admission of asthmatic patients in the hospital was not significant (p = 0.203) (Table 3). Out of the 19 admitted patients, six had CHD, but the effect of CHD on the admission of asthmatic patients in the hospital was not significant (p = 0.243) (p > 0.05) (Table 3).

**Table 3:** Association between comorbidities of asthma patients and admission in hospital. DM = diabetes mellitus; HTN = hypertension; GERD = gastroesophageal reflux disease; OSA = obstructive sleep apnea; CHD = congenital heart disease

Comorbidities	Number of admitted patients with comorbidities (Total = 19)	Percentage	P-value
DM	3	6.8 %	0.060
HTN	2	4.5%	0.014*
GERD	6	13.6%	0.05^*^
OSA	2	4.5%	0.203
CHD	6	13.6%	0.243
Total	19	43.18%	

Furthermore, 363 asthmatic patients visited ER, of whom 229 were males and 134 were females. Apparently, male asthmatic patients visited ER more compared to females, which was not significant (p = 0.752), thus we can say that there is no association between the gender of asthmatic patients and ER visits. More male patients visiting the ER is just by chance and not statistically significant.

Moreover, out of the 363 asthmatic patients, 26 had allergic rhinitis, and 53 patients had eczema. Apparently, male asthmatic patients had more atopic diseases compared to females, but for allergic rhinitis (p = 0.618) and eczema (p = 0.493), thus we can say that there is no association between the gender of asthmatic patients and the chance of having atopic diseases. More male patients having atopic disease is just by chance not statistically significant. In addition, out of the 362 asthmatic patients, 26 had allergic rhinitis, and 53 had eczema. Moreover, 10 patients had both eczema and rhinitis. The significance value for both variables was 0.002, which is highly significant, thus we can say that there is statistical evidence of a strong association between eczema and allergic rhinitis occurring simultaneously in asthmatic patients.

As shown in Table 1, out of the 363 asthmatic patients who visited the ER, 123 used steroids for asthma, and 240 patients did not use steroids. As shown in Table 4, patients who used steroids less frequently visit ER compared to those who did not use steroids (p = 0.000), which is highly significant, thus we can say that there is statistical evidence of a strong association between steroid use and decrease frequency to ER visit in asthmatic patients.

**Table 4:** Association between steroid use by asthma patients and the number of visits to the emergency room.

Emergency visit	Steroid use		Total	P-value
	Yes	No		
One visit	39	146	185	P = 0.000^*^ (p < 0.05)
	21.1%	78.9%	100%	
Two visits	18	45	63	
	28.6%	71.4%	100%	
Three visits	17	15	32	
	53.1%	46.9%	100%	
More than three visits	49	34	83	
	59.0%	41.0%	100%	
Total	123	240	363	
	33.9%	66.1%	100%	

Finally, asthmatic patients who were using steroids were found to be night symptoms free. As 91 patients, out of 123 who were using steroids, were not having night symptoms (p = 0.000). Thus, steroids can have a positive effect on reducing or inhibiting night symptoms, as shown in Table 5.

**Table 5:** Association between steroids use by asthma patients and night symptoms.

Parameter		Night symptoms		Total	P-value
		Yes, n (%)	No, n (%)		
Steroid use	Yes	32	91	123	P = 0.000^*^
		26.0%	74.0%	100%	
	No	23	216	239	
		9.6%	90.4%	100%	
	Total	55	307	362	
		15.2%	84.8%	100%	

## Discussion

It is a well-known fact that bronchial asthma under the age of 14 has a male gender predominance, as shown in multiple studies [21]. The study demonstrates that 63.1% of those who had an asthma attack were males. While there is no clear evidence why there is a male predominance when it comes to asthma, it is thought to be related to multiple factors, including social, environmental, and genetic factors.

Asthma exacerbation is an acute or subacute progressive worsening of asthma symptoms that has a huge negative impact on both the child’s health and the caregiver of that child as well. Despite advances in asthma control and the establishment of guidelines specifically for pediatric asthma, acute flares still occur and lead to significant morbidity in the child’s general health [22]. In the Asia-Pacific region, a survey conducted in 2006 showed persistently poor control in asthmatic children, which was reflected by the rate of ED visits as 19% of ED pediatric visits were due to URTI accompanied by asthma exacerbation [23]. Our study showed that 51% of those who had experienced an asthma attack previously visited the ED one time, and 17% of asthmatic patients had visited the ED at least two times. This could be attributed to many hypothesized factors, including poor control of asthma among Saudi pediatric patients, poor medication adherence, continuous allergen exposure, and the presence of medication-resistant asthma. The frequent ER visits clearly show that we need to spend more time educating patients or their caregivers about the nature of the disease and the various available ways to control such a disease.

The presence of other comorbidities with bronchial asthma has been reported to increase the occurrence of asthma exacerbation among the pediatric population. GERD-associated obesity has been linked to asthma exacerbation as the reflux leads to the inflammation of the airways [24]. Our study also found a significant association between asthma exacerbation and GERD. So, clearly, the control of GERD is an essential part of asthma management in those who have a concurrent GERD with bronchial asthma. Moreover, allergic rhinitis has been linked to asthma exacerbation as it was shown in previous studies that patients with uncontrolled severe bronchial asthma have a coexisting nasal disorder, commonly allergic rhinitis or chronic rhinosinusitis [25]. The study showed that 26 patients out of 363 patients with bronchial asthma have coexisting allergic rhinitis, with males being more affected. The occurrence of asthma along with allergic rhinitis and eczema was noticed in multiple studies, one of them showed that 22.6% of patients with bronchial asthma have both allergic rhinitis and eczema [26]. We also found statistical evidence of a strong association between eczema and allergic rhinitis occurring simultaneously in asthmatic patients.

A study evaluated the effect of inhaled steroid use on the prevalence of asthma-related hospitalization and ED visits. The study showed that patients who were on steroid therapy were less frequently visiting the ED when compared to patients who were not using steroid therapy for their bronchial asthma [27]. Our study showed a similar finding, as 123 out of 363 patients with asthma exacerbation were using steroids compared to 240 patients out of 363 who were not using steroid therapy and developed an asthma exacerbation. So, clearly, steroid therapy decreases the rate of ED visits. Regular use of inhaled steroid therapy is a well-known effective way to the treatment of persistent asthma symptoms during sleep (nocturnal asthma). It was reported in multiple studies that people who use steroid therapy have less frequent asthma symptoms during sleep [28]. Similarly, in our study, we found that people who were using inhaled steroids to treat asthma were less likely to develop night symptoms, as shown in Table 5.

Acute asthma exacerbation should be distinguished from poor asthma control. Patients with asthma exacerbation will present with progressive shortness of breath, chest tightness, coughing, and/or wheezing. On the other hand, poor asthma control usually presents with a nocturnal variability in airflow and is a feature that is sometimes not present in acute exacerbation [29]. In our study, we found that most asthma exacerbation patients presented to the ED with cough, shortness of breath, and/or fever. Fever was reported as a sign of asthma exacerbation; possibly because 89.3% of our patients had a history of URTI concurrent with the asthma exacerbation. To our knowledge, asthma development is influenced by the interactions among genetic factors, environmental, and behavioral factors [30]. The genetic role brings us to talk about the importance of family history in the development of asthma, as it is a well-known fact that a family history of bronchial asthma and atopy predisposes the child to develop asthma early in life. This study showed that 20.9% (n = 79) of patients had a family history of bronchial asthma and/or atopy. Climate change during different seasons is known to affect children’s health and allergic diseases turn out to be at the front row of the effects of seasonal variation in addition to infectious diseases. As a result, climate change imposes children to develop respiratory infections and exacerbates allergies. In our study, we found that the most common season for asthma exacerbation in children was winter followed by the fall season. In winter and fall, cold and dry air is associated with an increased risk of respiratory infections. Moreover, in the winter season, there is a higher risk of exposure to molds, dust mites, and indoor pollution caused by wood-burning stoves. This cold dry air irritates the respiratory airways leading to the constriction of the respiratory muscle, and, eventually, the development of URTI and asthma exacerbation.

This study had some limitations which included being conducted in a single center. Moreover, this study was conducted in one region of Saudi Arabia, Riyadh. A relatively small sample when considering a huge common disease such as bronchial asthma is also one of the limitations.

## Conclusions

Asthma is a common pediatric respiratory disease that could be a burden if not controlled well. Unfortunately, the frequency of hospital admissions and pediatric ER visits due to asthma exacerbation is increasing. Comorbidities such as obesity and GRED play a significant role in asthma control. This study has some limitations such as the duration of steroid use was not calculated, and a more comprehensive, nationwide, large study should be conducted to address the presented issue clearly. Future studies are recommended to investigate the relationship between asthma and obesity from a laboratory and clinical point of view.
